# Reducing risk and improving maternal perspective-taking and empathy using virtual embodiment

**DOI:** 10.1038/s41598-018-21036-2

**Published:** 2018-02-14

**Authors:** Catherine Hamilton-Giachritsis, Domna Banakou, Manuela Garcia Quiroga, Christos Giachritsis, Mel Slater

**Affiliations:** 1 School of Psychology, University of Birmingham, Birmingham, UK; 2 Department of Psychology, University of Bath, Bath, UK; 3 Experimental Virtual Environments for Neuroscience and Technology (EVENT) Laboratory, Department of Clinical Psychology and Psychobiology, University of Barcelona, Barcelona, Spain; 4 Institute of Neurosciences, University of Barcelona, Barcelona, Spain; 5 Research Directorate, British Maritime Technology Group Ltd, London, UK; 6 Institució Catalana de Recerca i Estudis Avançats (ICREA), Barcelona, Spain; 7 Department of Computer Science, University College London, London, UK; 8 Present Address: Pontificia Universidad Católica de Valparaíso (Escuela de Psicología), Valparaiso, Chile

## Abstract

The ability to perspective-take (cognitive awareness of another’s state) and empathise (emotional/affective response) are important characteristics for sensitive, co-operative and constructive parenting, which assists in developing adaptive functioning for children. For the first time, immersive virtual reality was used to place parents in the position of a child in order to assess impact on perspective-taking and empathy. This novel study was conducted with 20 non-high risk Spanish mothers (a pilot study with 12 mothers is reported in supplementary files). Mothers were virtually embodied as a 4-year-old child, experienced from the first-person perspective and with virtual and real body movements synchronised. They interacted with a ‘mother avatar’, which responded either in a Positive or Negative way. Participants reported a strong body ownership illusion for the child body that led to cognitive, emotional and physical reactions. Experiencing negative maternal behavior increased levels of empathy. In addition, the Negative mother led to increased feelings of fear of violence. Physiological data indicated greater stress in the Negative than Positive condition. Although further research is required to assess the effectiveness of such methods, any improvement in empathy that leads to a change in parenting behavior has the potential to impact on developmental outcomes for children.

## Introduction

Empathy deficits have been associated with a range of disorders and offending behaviors, most notably risk of offending^1^, psychopathy^2–4^, sexual offending^5^ and autistic spectrum disorders^6,7^. Although the relationship between empathy and parenting difficulties is less widely researched, arguably empathy has a significant role to play given the importance of both recognising and feeling a child’s emotions in being able to sensitively parent^8^. Positive warm parenting assists in developing adaptive functioning for children; a nurturing parent has the ability to demonstrate empathy towards the needs of their child and place those needs in high regard. In contrast, negative parenting can cause long-term negative outcomes for children. Parents with low empathic awareness of their child’s needs often have difficulty helping children meet those needs - normal developmental needs are seen as the child being ‘annoying’. Hence, developing empathy in parenting has the potential to improve outcomes for children and young people. This is particularly true for families where there is risk of child maltreatment (CM).

## Parenting and child maltreatment

Vast numbers of children are abused and neglected by their own family each year^9,10^, with a high rate of recurrence^11^. There is a significant impact on mental health and other provision for victims, interventions for abusers, as well as costs for public child care, criminal justice, social work and specialist education^12,13^. Whilst it has been shown that some people demonstrate remarkable resilience to CM^14^, for others the effects of such victimisation can be seen both in childhood and in adulthood in a variety of domains, including physical, psychological, social and interpersonal relationships^15–17^. Notably, when combined with risk factors (e.g., history of mental illness or depression), poor parenting styles (e.g., negative attributions) have been identified as mediating the likelihood of a maltreated child becoming a maltreating parent (intergenerational cycle of maltreatment^18^). In contrast, realistic expectations and attributions can reduce risk and improve outcomes for children.

Helping a parent remember how it feels to be dependent on others to meet your needs has the potential to be a crucial step in developing more positive parenting responses and improving outcomes for children. However, whilst some things can be taught through parental education (e.g., children’s developmental stages, realistic expectations), empathy and perspective-taking are seen as difficult to teach. Indeed, this difficulty has been noted in work with sexual offenders^19^.

Thus, if the ability to perspective-take and empathise are important characteristics for positive parenting but are hard to teach, we are left with two questions: how can we help parents understand what it feels like to be a child? If we help parents understand what it feels like to be a child, can we improve their empathy and parenting?

## Empathy and perspective-taking

It is important to distinguish between the different aspects of awareness of another’s state, which can be both affective and cognitive. Empathy is described as the ability to identify with and understand another’s situation, feelings and motives (emotional/affective), whilst perspective-taking (PT) is the ability to see things from another’s point of view (cognitive), i.e., recognition of, but not necessarily feeling, an emotion. For example, it is widely argued that psychopaths are able to PT and recognise the emotion of their victim, but are unable to demonstrate any emotional response of their own; similarly, they can describe emotion, but not feel it (as highlighted in^20^, entitled ‘*They know the words but not the music’*). In contrast, an individual might be poor at PT but able to empathise once they are helped to recognise the emotion, and it is this situation in which it may be possible to enhance parenting. Thus, there is potential to help parents learn to recognise their child’s emotions which may, in turn, impact on their ability to empathise.

One difficulty with working with families where abuse and/or neglect are occurring is how to achieve your aims in a safe, constructive environment. Immersive virtual reality (IVR) provides a possible forum in which parents could be reminded how it feels to be a small child (e.g., limited ways of asking to have needs met, physical size difference), with the potential to encourage perspective-taking (PT) and empathy. Although therapeutic techniques exist (e.g., ‘empty chair’, where you take the seat of the other to facilitate understanding of their view other), IVR could potentially increase the effectiveness of this method and make it accessible also to those who struggle to do such work imaginatively, as illustrated in^21^.

Past research has shown that empathy and helping behavior can be enhanced and promoted through the use of prosocial media, console gaming and virtual reality^22–26^. Studies have examined how behavioral tendencies in a virtual environment can be predicted from dispositional measures of compassion and empathy^27^, or how participants’ empathic feelings towards animated characters can be enhanced through different simulated virtual scenarios^28–30^, targeting, for example, bullying behaviors^31,32^ or training with patients^33,34^.

## Embodiment in immersive virtual reality

IVR has been successfully used to generate illusions of ownership over whole virtual bodies of different gender, race or even age^35–38^, with changes in cognition and perception. For example, embodiment of participants in a dark-skinned virtual body seen from first-person perspective (1PP) led to a reduction of implicit racial bias^37^, which was sustained over time^39^. In relation to children, previous research has shown that virtually embodying adults into the body of a young child leads to changes in perception of sizes in the environment and implicit self-identification, with a shift of implicit attitudes about oneself towards being childlike that does not occur when the body is adult size^35,40^.

Therefore, the aim of this study was to establish whether embodiment of mothers into the body of a young child and engaging in an interaction with a virtual mother facilitates perspective-taking and empathy. To address this question, a pilot study was conducted with twelve non-high risk mothers (results reported in supplementary file), followed by a main study with another twenty non-high risk mothers (reported below). Participants were embodied in a child body while experiencing either a positive (Positive) or negative (Negative) interaction with a virtual mother. In order to induce a level of subjective body ownership, the technique of visuomotor synchrony was used (i.e., full body motion capture suit and field-of-view head-tracked stereo head to enable viewing of virtual body when looking down towards their real body and in a virtual mirror). Specifically, the research questions were:

1. Is there evidence of an illusion of body ownership with the virtual child body and does the strength of the illusion differ between the two virtual interactions?
2. Does the IVR system reproduce in participants the feelings of a young child, hence evoking perspective-taking (PT)?
3. Are participant experiences in the IVR system affected by whether the maternal-figure interaction is positive or negative?
4. Is this IVR experience associated with changes in levels of empathy?

## Methods

### Ethical considerations

The study was approved by the University of Birmingham STEMM ethics committee (ref. ERN-12-1542) and by Comissió Bioètica of Universitat de Barcelona (IRB00003099). The study was also carried out in accordance with relevant codes of conduct. i.e., the British Psychological Society and the Heath and Care Professions Council. Ethical considerations included gaining informed consent, right to withdraw, limits of confidentiality and sources of support, if required. The person undertaking the pre/post interviews and assessment was a qualified clinical psychologist, in order to ensure that any participants who became distressed received appropriate support. Following completion of the second phase, participants were debriefed. All participants were contacted by email one week later to make sure there were not any undesired ‘after-effects’ from the IVR exposure.

### Experimental Design

#### Pilot study

A pilot study was conducted to test the technical aspects of the system prior to the main study (see supplementary information). Following this, the Mind in the Eyes test was added for a measure of general empathy in the main study.

#### Main study

**Figure 1:**
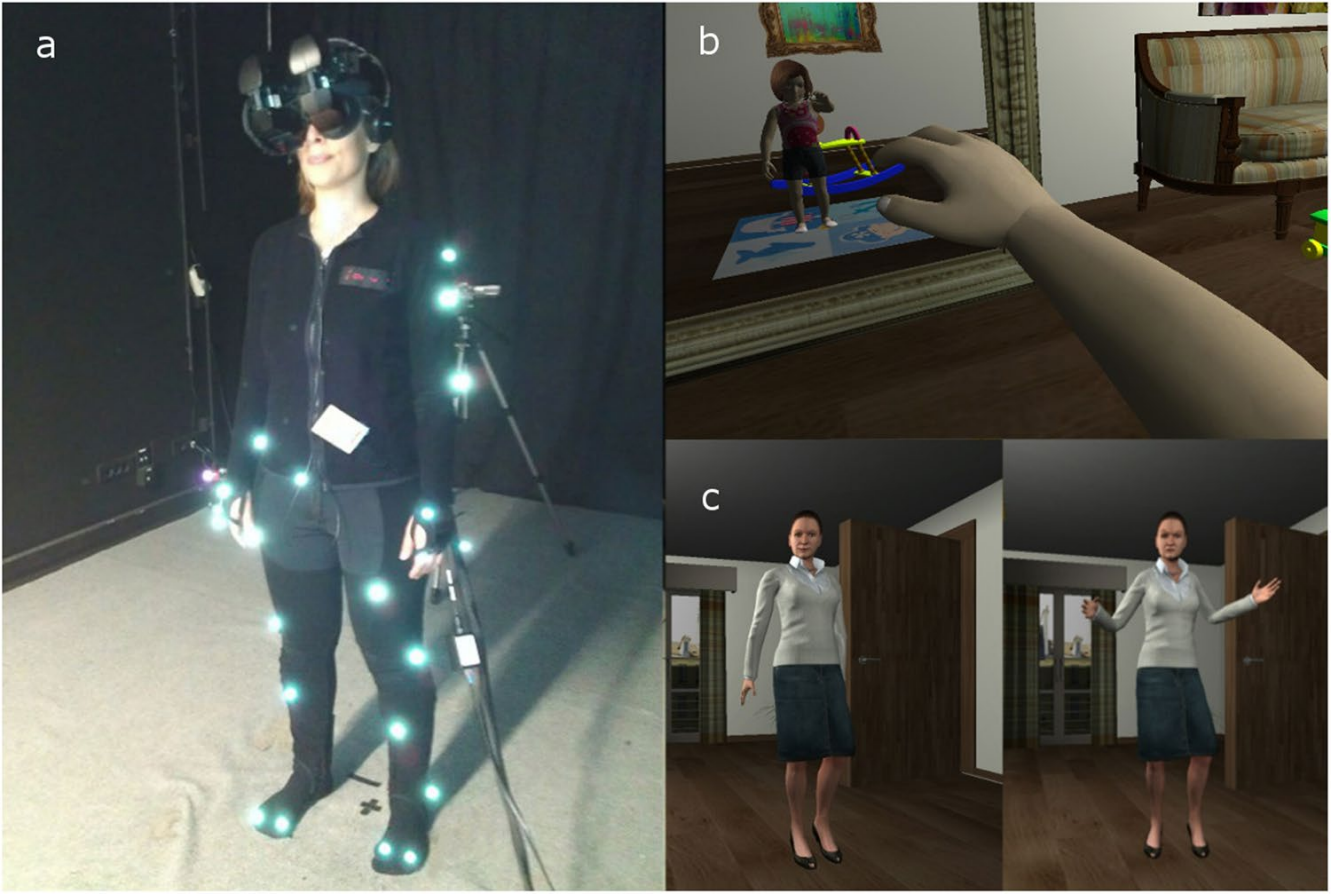
Experimental setup. (**a**) Full body motion-capture suit/head-mounted display. (**b**) Body of the participant substituted by a child virtual body and visible in a virtual mirror. (**c**) Two different interaction conditions with a virtual mother: Positive (left) or Negative (right).

This was a within-groups counter-balanced design with a single binary factor ‘Behavior’. Participants were randomly allocated to one of the two designed groups: experiencing the Positive mother first and then the angry mother (Condition ‘Positive’), or the angry mother first and then the Positive (Condition ‘Negative’; Fig. 1c). The two sessions were separated by two days; measurements were identical for both sessions.

### Participants

Twenty-one Spanish mothers aged 31 to 47 years (mean age 39.3, SD = 4.05, SE = 0.9) with normal or corrected vision took part; one dropped out due to simulation sickness so data are reported on 20 participants. All participants were the biological mother of at least one child (total of 30 children, M = 1.51 children, range 1–3); children’s ages were 6 months to 15 years (M = 6.23 years, SD = 3.36, SE = 0.61). All participants reported little or no prior experience of virtual reality. (Experimental groups were comparable across a number of variables; Supplementary Table S1).

Exclusion criteria were epilepsy, use of medication, recent consumption of alcohol, significant back or neck difficulties, intellectual disability and mental health difficulties. Participants were paid €50 for participating. For ethical reasons, prior to working with parents who may be seen as high risk for CM, the study focused on a community sample of mothers without known parenting difficulties. Given the differences both in male/female empathy and in parenting, a decision was made to exclude fathers from the initial study.

### Procedures

Mothers were recruited through posters, e-mail, word of mouth and an online forum. An information sheet outlined limits of confidentiality, right to withdraw, safety regarding driving post-experiment and where to access support if necessary. Written informed consent was obtained and pre-measures completed (in Spanish or Catalan). Participants were fitted with the head-mounted display (HMD), the body-tracking suit and physiological data recording devices. The view through the HMD was individually calibrated.

During experimental phase one (five minutes), participants entered a virtual living room, which included a virtual mirror and children’s toys. Their body was substituted by a 4-year-old virtual child body, seen from first-person perspective (1PP). Head and body movements were mapped in real time to the virtual body, seen both by looking directly down at their real body and in the virtual mirror (Fig. 1b). Following pre-recorded instructions, participants performed a set of stretching exercises to explore the capabilities of the virtual body, then continued moving freely whilst describing what they saw.

In phase two, participants were asked to standstill/relax for baseline physiological data. During phase three, the virtual mother entered and initiated conversation with the participant (Fig. 1c) – either Positive or Negative depending on condition. The mother avatar then left the room, the participant was alone for 30 seconds before the experiment finished. Post-experiment interview and questionnaires followed (Table S2). Two days later, the second session followed the same procedure with the remaining condition (Positive/Negative).

### Measures

Psychometric and qualitative data were collected pre, during and post experiment; heart rate and galvanic skin response during. Data included demographic details, the Parenting Scale Inventory to assess discipline practices^41^ (PSI), the Mind in the Eyes test of empathy^42^, the Adult Adolescent Parenting Inventory – version 2^43^ (AAPI-2) to assess parenting and child rearing attitudes (including a measure of empathy), a post-experience questionnaire and a semi-structured interview (Tables S2 and S3). As noted, empathy was therefore measured in two ways: Mind in the Eyes test (general empathy) and AAPI subscale (parenting empathy). [Further information is given in Suplemmentary Information and Supplementary Movie S1].

### Data availability

The datasets generated during and/or analysed during the current study are available from the corresponding author on reasonable request.

## Results

### Body ownership and agency

**Figure 2:**
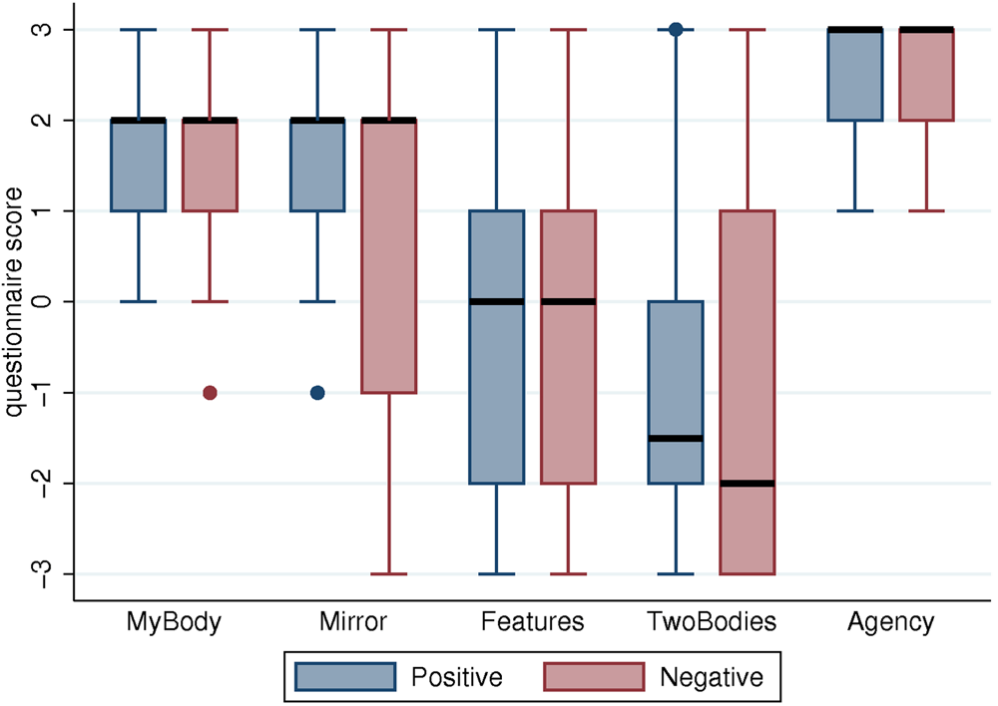
Extent of body ownership and feelings of agency (black horizontal line: median; box: interquartile range; whisker: max (lowest score, median−1.5IQR) and min (highest score, median + 1.5IQR).

The extent to which participants felt body ownership over the child virtual body and agency over its motor actions was assessed by a questionnaire after each experimental condition (Table S2). Scoring was on a Likert scale (−3 to +3), with two questions on body ownership (*MyBody; Mirror*), two related control questions (*Features; TwoBodies*) and one on agency about the virtual body (*Agency*). Figure 2 shows that participants tended to affirm the illusion of ownership and agency, with no significant difference between the two ‘Behavior’ conditions (i.e., Positive or Negative mother) nor order of condition.

Overall, body ownership and agency were high (median scores 1–3). The control questions (*Features; TwoBodies*) had medians between −2 and 0, and mostly their interquartile ranges (IQR) do not overlap those of *MyBody* and *Mirror*. The most overlapping IQRs are between *Mirror* and *Features* for the Negative condition. A Wilcoxon matched-pairs signed rank test rejects the hypothesis of equal medians (z = 2.782, P = 0.005). Hence if this difference is significant all of the other paired tests (*Mirror* or *MyBody* against *Features* or *TwoBodies*) will also be so. Although there are no differences between conditions with the illusion questions, *MyBody*, *Mirror* and *Agency* have greater scores than the control questions (*Features*, *TwoBodies*).

#### Emotional response

**Figure 3:**
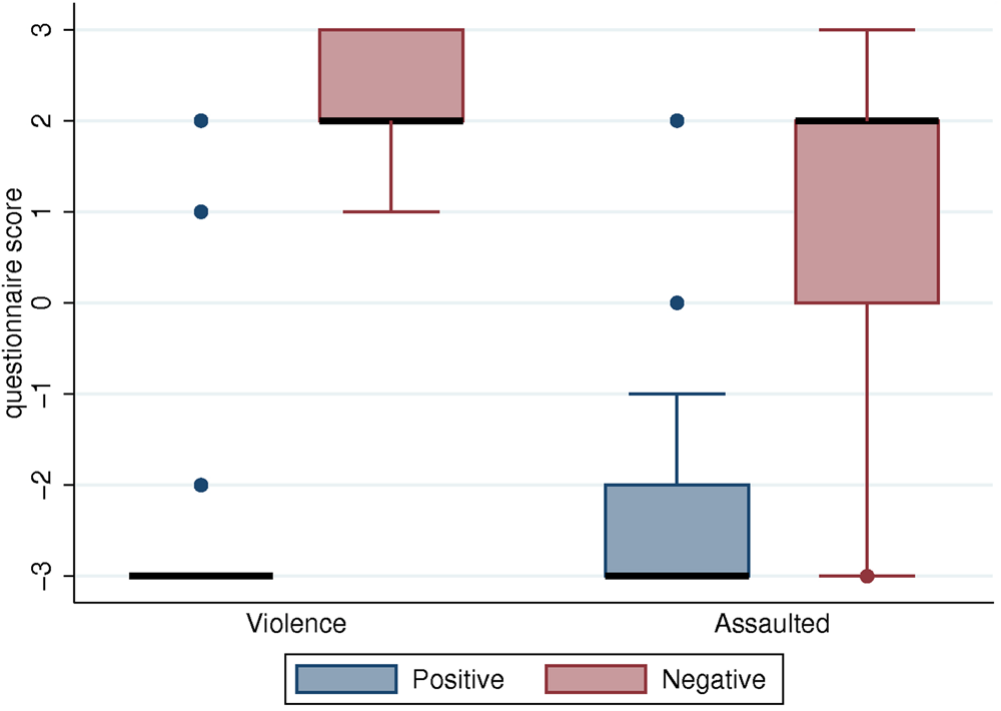
Level of violence (*Violence*) and extent to which participants felt they might be physically assaulted (*Assaulted*) by the virtual mother.

Two important variables were the extent to which participants felt that the scene they experienced was violent (*Violence*) and how much they experienced the possibility that they might be physically assaulted by the virtual mother (*Assaulted*). There were very low scores for both *Violence* and *Assaulted* (with some outliers) with the Positive mother, but high scores with the Negative mother (Fig. 3). Hence the participants experienced strong body ownership over the child body and their experience was influenced by the virtual mother.

### Parenting Style and empathy

#### Parenting Scale (PSI)

Mean scores were comparable to published control group scores for Laxness (i.e., little limit setting) and clinical group means for Over-reactivity and Total score (Table 1), but were higher for Verbosity (i.e., amount of talking). This outcome could reflect cultural variations, but the Verbosity scale also has the lowest internal consistency.

**Table 1:** Parenting styles of participants and normative data for comparison (N = 20).

	Spanish group (N = 20)		Norms*	
			Clinical (N = 26)	Control (N = 51)
	Mean (SD)	range	Mean (SD)	Mean (SD)
Mother’s age	39.2 (SD = 4.0)	31–47	29.6 (6.7)	31.7 (3.9)
Laxness	2.6 (0.5)	1.6–3.5	2.8 (1.0)	2.4 (0.8)
Over-reactivity	2.9 (0.5)	2.0–4.1	3.0 (1.0)	2.4 (0.7)
Verbosity	4.3 (0.6)	3.0–5.1	3.4 (1.0)	3.1 (1.0)
Total score	3.2 (0.3)	2.6–3.9	3.1 (1.7)	2.6 (0.6)

*O’Leary, Arnold, Wolff, & Acker (1993).

#### Empathy (AAPI-2)

 Pre-intervention score for empathy in parenting was already in the low risk *category* and remained so post-intervention. However, looking at the *score*, post-intervention was significantly higher than pre-intervention (i.e., even lower risk), rising from 8.1 to 9.0 (t = −2.538; p = 0.02; Table 2).

**Table 2:** AAPI-2 scores pre and post scenarios (N = 20)*.

Construct	Pre Mean (SD)	Post Mean (SD)	t	Significance
**Construct A** Expectations of Children	8.15 (1.2)	8.3 (1.3)	−0.616	0.545
**Construct B** Parental Empathy towards Children’s Needs	8.10 (1.5)	9.00 (1.3)	−2.538	**0**.**02**
**Construct C** Use of Corporal Punishment	8.30 (1.8)	8.65 (1.5)	−1.129	0.273
**Construct D** Parent-Child Family Roles	9.6 (1.0)	9.2 (1.5)	1.435	0.163
**Construct E** Children’s Power and Independence	7.10 (2.1)	6.20 (2.3)	2.100	**0**.**049**

*Range 1–10; high score is positive, low risk; low score (3 or under) is negative, high risk.

Construct E showed a significant change from valuing power and independence in children (7.10; medium risk) towards a greater tendency to restrict power and independence (6.20; medium risk; t = 2.100, p = 0.049). This outcome seems to reflect extreme changes in scores by a few participants (e.g., very low risk pre to very high risk post), but requires further investigation.

#### Order effect

**Figure 4:**
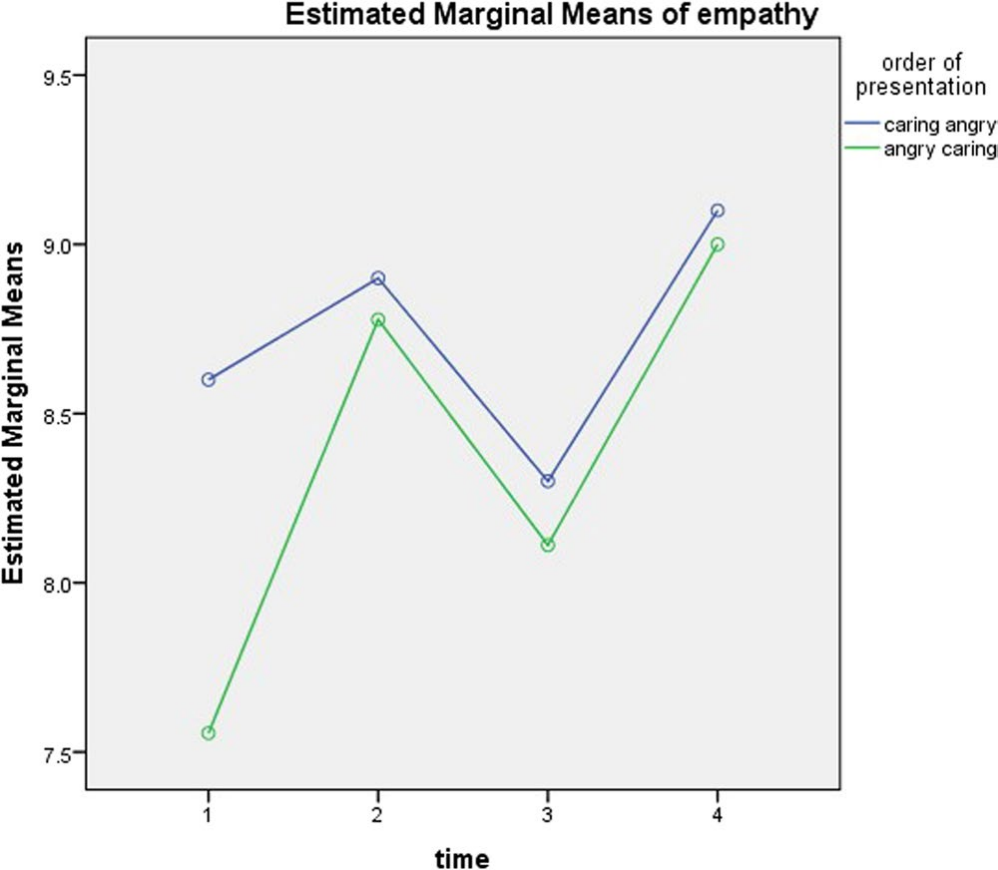
Changes in empathy over time (N = 19; F (3) = 3.673, p = 0.018 (assumption of sphericity met).

There were changes in empathy over time, F(3) = 3.673, p = 0.018 (assumption of sphericity met), but no interaction between time and order F(3) = 0.846, p = 0.475 (Fig. 4). Order was not significant, F(1) = 0.427, p = 0.522. Pairwise comparisons (LSD adjustment for multiple comparisons) found significant differences between times 1 and 4 (p < 0.05) plus 3 and 4 (p < 0.05).

### Parenting styles and AAPI-2 scores

There were a number of significant negative correlations between parenting beliefs/styles and AAPI-2 parenting, including empathy (Table 3). When order of presentation was not considered, before any experience in the system, lack of empathy was negatively correlated with laxness and total score (combined over-reactivity, laxness and verbosity). However, following first exposure (whether Positive or Negative), no correlations were found between parenting style and behavior. Notably, some were present again both pre- and post- *second* exposure. In addition, lack of empathy was again correlated with high laxness (r = −0.439; p = 0.05).

**Table 3:** Significant correlations between types of parenting behavior (AAPI-2) and parenting styles (PSI) for all participants (N = 20)*.

			r	P
Pre- first exposure (either Positive or Negative)	Inappropriate expectations:	Over-reactivity	−0.457	0.037
	Lack of empathy:	Laxness	−0.557	0.009
		Total score	−0.544	0.011
	Physical punishment:	Laxness	−0.604	0.004
		Over-reactivity	−0.463	0.035
		Total score	−0.598	0.004
Post-first exposure	—			
Pre-second exposure (either Positive or Negative)	Physical punishment:	Laxness	−0.598	0.007
		Over-reactivity	−0.463	0.046
		Total score	−0.541	0.017
Post-second exposure	Lack of empathy:	Laxness	−0.439	0.053
	Physical punishment:	Laxness	−0.589	0.006
		Over-reactivity	−0.463	0.04

*The second exposure occurred two days after the first exposure.

When order of presentation was considered, Positive-Negative had correlations between empathy and parenting styles (laxness, over-reactivity and total; range r = −0.665 to −0.819; p < 0.05 to < 0.005) at pre-first exposure with none at post-first or second exposure. For Negative-Positive, relationships were found with risk of physical punishment at each of the four assessment points (range r = −0.597 to −0.793; p = 0.05 − < 0.01), whilst lack of empathy was only negatively correlated at the post second exposure (i.e., positive) mother with laxness (r = −0.695, p < 0.05).

### Mind in the Eyes Test

**Figure 5:**
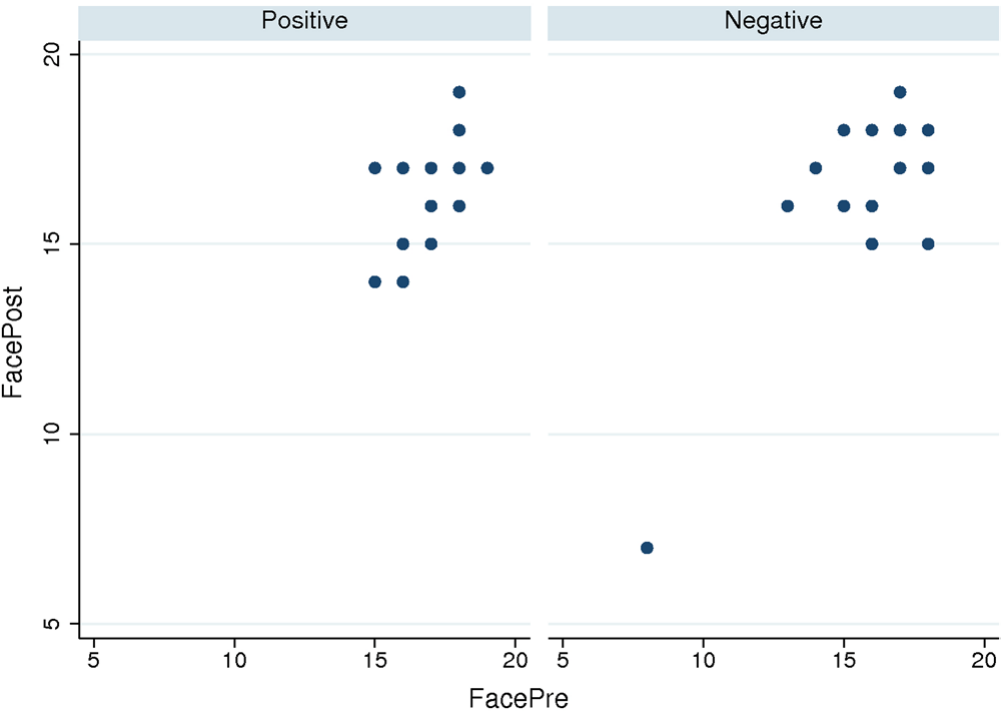
Scatter diagram of FacesPost on FacesPre.

**Figure 6:**
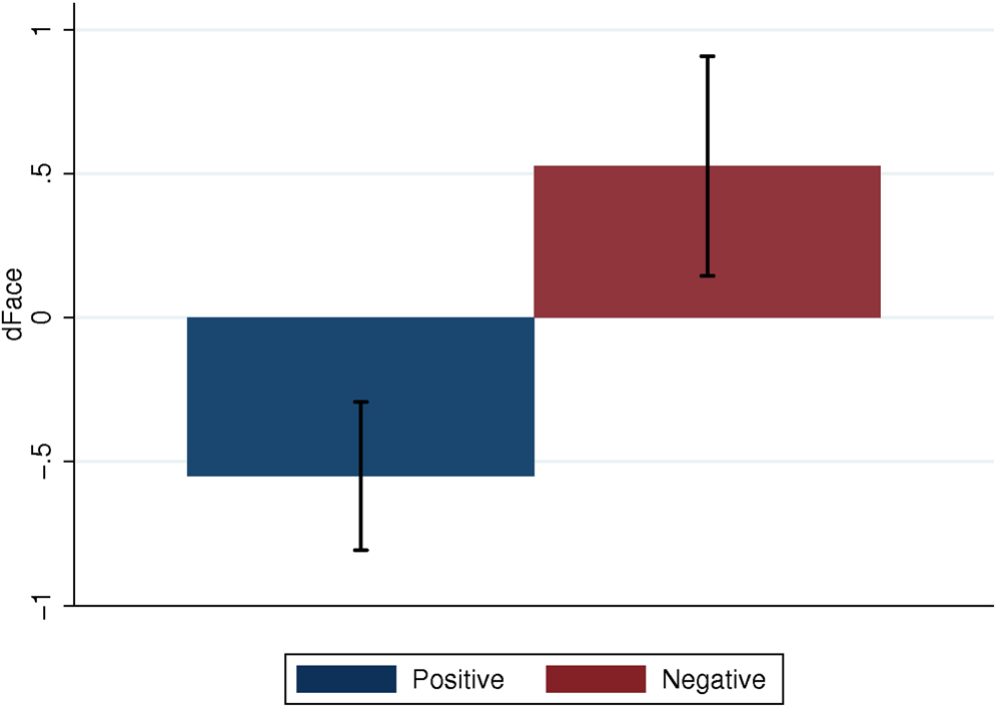
Means and standard errors of dFaces (FacesPost – FacesPre) showing correct emotion classification after and before the VR experience. (Higher values indicate increase in emotional recognition).

This task (pre- and post- each intervention) involved describing the emotional state of a person based on only an image of their eyes (see Supplementary Information), with images appearing in random order. It is a standard measure of empathy, not necessarily related to parenting. Correct classification pre and post intervention was measured. The variable of interest was dFaces = FacesPost – FacesPre, with greater values indicating an increase in emotional recognition. Figure 5 shows one extreme outlier (Fig. 5), which was removed and means and SEs of dFaces calculated (Fig. 6). Interacting with the Negative mother increased the number of correct classifications, but this did not occur for those who experienced the Positive mother. A mixed effects ANOVA, with fixed effects Behavior (Positive = 0, Negative = 1) and random effects over the individuals shows that the dFaces is greater for the Negative than the Positive mother (P = 0.018, 95% CI for the coefficient of Behavior 0.19 to 1.97), using the robust option to allow for departures from model assumptions. (Without this option the results are almost identical, P = 0.014, CI: 0.21 to 0.94, and including the outlier again the results hardly change, P = 0.023 with the robust option and P = 0.020 without it). This suggests that, according to this measure, experiencing the Negative mother was associated with an increase in empathy compared to the Positive.

### Analysis of Physiological data

#### Skin Conductance Response

The numbers of Skin Conductance Responses (SCR) were recorded during the baseline (SCRBase) and the experience (SCRExp). The amplitude of each SCR was also recorded (SCRAmpBase, SCRAmpExp); dSCR = SCRExp - SCRBase, and dSCRAmp = SCRAmpExp - SCRAmpBase. Of interest was whether the numbers and mean amplitudes are greater for the Negative than for the Positive mother. Supplementary Figure 2a shows dSCR by Behaviour; although the number of SCRs is greater in the Negative Behaviour condition (Negative), a Poisson log-linear mixed effects model allowing for robust standard errors shows that the difference has p = 0.56). Supplementary Figure 2b shows the mean SCR amplitudes, suggesting that the mean is greater for the Negative than the Positive mother. Mixed effects ANOVA supports this. The Negative mean is greater than the Positive (p = 0.002) and the 95% CI for the coefficient of Behaviour is 0.06 to 0.26.

#### Heart Rate Deceleration

The electrocardiogram was used to compute heart rate deceleration (HRD) during the experimental period while the mother virtual character approached the participant. Greater deceleration indicates greater distress or disgust. Supplementary Figure 3 shows indeed that the slope is steeper for the Negative mother than the Positive. Although the standard error is high in the case of the Negative mother, the mixed effects regression does not show a significant difference (p = 0.28).

### Participants’ reflections

Responses from the brief, semi-structured interview across the two studies identified general themes about participants’ experiences, which were consistent with other data, including:

- Feeling like a child, with frequent comments about size difference: “*It was a very strong experience seeing myself there, small and with another adult person*”.
- Better understanding how their sons/daughters feel.
- Fear of the mother and of being hit: “*I had more fear*”; “*I felt like crying*”
- Experiencing the negative mother as dominating; hence ‘shutting off’ because she would not listen:

- ○“*you stay there, like paralysed”;*
- ○“*I felt more comfortable being silent, just observing her… it wouldn’t make things better but worse”*.
- ○“*I had to take a few steps back, move backwards”*

- Order made a difference. When the angry mother came first, participants generally did not trust the ‘Positive’ mother: “*Well I think maybe I remembered the previous situation so I couldn’t trust her I was thinking that in any moment she was going to scold me again*”
- Almost all said the experience made them think about their own parenting: “*It is true that these last days I have been thinking about this*”.
- Some noted the difficulty of maintaining change (“*sometimes there are situations that go beyond your control … I think that will continue to happen to me, that I will have bad days*”), but others noted it had made them more mindful and that might continue (“*I don’t know for how long but in the immediate for sure, that I will be more aware, because these last days it has happened to me*”).

## Discussion

The primary purpose of this study was to determine whether embodiment techniques can be used for evoking empathy and perspective-taking in mothers. To consider this, it was first necessary to demonstrate that there was an illusion of body ownership and different impacts of negative versus positive interactions in the virtual environment. The results demonstrated that the majority of participants did feel immersed in the environment, felt ownership of movements and child-like, and their reactions occurred at cognitive, emotional and physical levels.

This subjective illusion of ownership of a virtual child body supports that found with different body forms^37,44,45^, including that of a child^35^. No significant differences were found for body ownership between the two interaction conditions. This result serves as a reference point, showing that there are no variations in the illusion of ownership between the two conditions that could account for the other findings. Furthermore, the results reveal that manipulating the two different interactions, introducing either a positive or negative maternal behavior, succeeded in a practical sense, as participants perceived the Negative mother as more violent and threatening, with a fear of potentially being physically assaulted. It is also supported to some extent by the physiological responses, with greater change SCR amplitudes from baseline during the interaction with the Negative mother compared to the Positive. In fact, all of the physiological responses point in the direction of greater stress in the Negative compared to Positive conditions, even though only one is statistically significant.

This is supported by the personal reflections on the experience, where participants reported feeling very much like a child and that it enhanced their perspective of being a child. So, it appears that the IVR environment assisted with perspective-taking in this group of mothers. Importantly, the data demonstrated that empathy levels changed, as assessed both for empathy for a child’s needs (AAPI) and more generally (Mind in the Eyes). Pre-post empathy scores (AAPI-2) significantly changed in a positive direction. The fact that the parents were not initially high risk reflects the participants’ recruited for this study. It was seen as important for ethical reasons to explore these concepts with non-high risk parents before applying the procedure to those parents identified as high risk of child abuse.

Interestingly, there was no interaction with order of presentation (i.e., which mother came first). This appears to suggest that the effect comes from experiencing both a positive and a negative mother avatar, rather than which comes first. Having said that, the verbal feedback from participants is that experiencing the Negative mother first affected how they viewed the Positive mother, i.e., with more suspicion and expecting anger. Therefore, the lack of order effect may have resulted from the way in which empathy was being measuring in this study. Indeed, if we look at the Mind in the Eyes test, which has been widely used across different groups and cultures^42^, the fact that participants performed better at assessing emotional states following their negative interaction with the virtual mother, supports the idea that the method improves feelings of empathy towards the child.

This finding is in line with several psychological interventions where patients are subjected to negative experiences, which can be distressing, temporarily, but which ultimately aim to improve their symptoms, and help overcome psychological problems. For example, in the case of phobias, treatment is largely based on exposure-therapy, which involves exposing the patient to the anxiety source in order to help them overcome their distress. Moreover, evidence suggests that patients suffering from post-traumatic stress disorder may benefit from imaginatively re-experiencing traumatic events, eventually leading them to the extinction of fear-related responses^46^.

Our results fit with a recent study^47^, where IVR was used to increase self-compassion in self-critical individuals. Participants delivered a compassion speech to a crying virtual child and then watched their speech, either embodied as that child or in a third person non-embodied condition. Being embodied as a child led to greater degree of self-compassion, whereas the non-embodied group had no significant changes. Similarly, participants in ref.^21^ were embodied a self-alike virtual body, describing a personal problem, or a Sigmund Freud virtual avatar, from which they offered themselves counselling. Whilst self-counselling was positively evaluated by participants, mood and happiness improved when the counsellor resembled Freud. Hence, although embodiment can be effective in improving self-compassion (providing additional support for the idea that PT can be similarly enhanced), seeing one’s own body from a different location only — literal perspective taking – might not be as efficient as originally thought. In our study, the embodiment as a child (not an adult) was important.

One unexpected finding was the significant shift in power and independence, but in a negative direction (i.e., parents were more likely to express views related to restricting independence post intervention). This may result from one or two participants who had an extreme shift (i.e., very low to very high risk) but requires investigation to establish the implications and/or why some people have this response. One option would be to consider this qualitatively, since it may be that the experience shifted the way in which those participants viewed the questions; for example, rather than reading the scenario as restricting independence, were they read as being more supportive and involved? However it is very important to consider why some individuals have this reaction before it is trialled with a high risk group.

In terms of future study, one factor to be taken into consideration is the ‘conversation’ taking place between the virtual mother and the participant, as well as the mother’s body language and facial expressions. Our study was based on a predetermined script – we aimed to keep the dialogue between the two conditions as close as possible, but also within a relatively neutral context – therefore, it would be valuable to examine in the future how variations in the script may affect the results. In addition, comparing IVR to different media-based techniques, such as watching the scenario on TV or a computer screen, would add further valuable knowledge to the effectiveness of our method. Interestingly, it has been shown that interventions that have been carried out using virtual reality exposure therapy, result in therapeutic outcomes comparable to those obtained using *in-vivo* exposure, or better^15–17^.

In conclusion, this study demonstrated that it was possible to use an IVR environment to enable mothers to take the perspective of a child and, in the short term at least, to create change in levels of empathy. Notably, the Negative mother had more impact on changes when assessing empathy generally and in self-report (not on the AAPI); thus, it suggests that the negative element of the experience is necessary. However, future research is necessary to consider a number of factors, including maintenance of increased empathy, impact on actual parenting behavior change and length of intervention. Indeed, there were indicators that some elements of parenting beliefs on the AAPI changed only temporarily but the changes in empathy did persist over time. Most importantly, it will be necessary to more fully explore the issues of power and independence before this approach could be developed with high risk parents. However, these are promising early findings – not least because these changes have occurred even with non-high risk parents – and clearly indicate that there is value in exploring these avenues further.

## Electronic supplementary material

Supplementary information

Movie S1
