# Supplementary material for: Reducing risk and improving maternal perspective-taking and empathy using virtual embodiment
Source: Sci Rep. 2018 Feb 14;8:2975. doi: 10.1038/s41598-018-21036-2 (PMC5813089; doi:10.1038/s41598-018-21036-2)
Supplement: Supplementary file 1 — Supplementary information [file 41598_2018_21036_MOESM1_ESM.pdf]

Reducing risk and improving maternal perspective-taking and empathy using virtual embodiment

Catherine Hamilton-Giachritsis, Domna Banakou, Manuela Garcia Quiroga,

Christos Giachritsis and Mel Slater

SUPPLEMENTARY INFORMATION

Materials and Methods

Environment. The experiment was conducted in a VR lab (width: 2.96 m, length: 3.4 m - back wall to curtain - height: 2.87 m). Participants were fitted with a stereo NVIS nVisor SX111 head-mounted display. This has dual SXGA displays with 76°H×64°V field of view (FOV) per eye, totalling a wide field-of-view 111° horizontal with 50° (66%) overlap and 64° vertical, resolution of 1280×1024 pixels per eye displayed at 60Hz. Head tracking was performed by a 6-DOF Intersense IS-900 device. The Optitrack full body motion capture suit uses 34 markers and is calculated with Motive software to track their movements (SFigure 1a). The infrared technology was implemented with a 12-camera truss setup by OptiTrack. Virtual models were modelled in 3D Studio Max 2010 and DAZ Studio V.3, with the virtual environment implemented on the Unity 3D platform.

The mother avatar. The mother figure was modelled to be a middle-aged female, speaking Castilian (the accent of the real actress who recorded the script). Her behaviour consisted of a number of multi-modal utterances, including pre-recorded speech, body and facial animation, which were triggered from a control panel by an experimenter. There were around 20 utterances in total prepared and pre-recorded including backups for unforeseen situations. Each utterance was a combination of speech (audio file) and synchronous lip-movements (facial animation). The animation composed of foreground behaviour, the multi-modal utterances, and background behaviour including gaze, which

ensured that the mother was mostly oriented towards the participant. She also maintained a conversational distance of 1.6 meters, by breaking this towards the end of the interactive conversation as she approached closer to the participant at 0.8 meters.

The mother's behaviour was modelled to reflect the personality of either a caring mother behaving in a proper way (Condition 'Positive') or a commanding parent behaving in an inappropriate way (Condition 'Negative'). In both cases she initiated the conversation with the participant by asking her questions with the respective tone of voice. In the Positive condition she appeared to listen carefully and showed her interest by leaning towards the participant, and smiling while looking at her, always replying in a soft voice. In the second case, she appeared to be very authoritarian, constantly avoided looking at the participant, and her tone of voice and facial expressions revealed an irritated mood. In each case the whole conversation lasted about 3 minutes. The system was 'fixed' in terms of parent avatar responses, therefore the parent responses had to account for a number of participant responses (for example, 'which toy do you like?'; 'that is a nice toy'). Furthermore, the complete conversation dialogues were intended to match as close as possible in both conditions and therefore consisted of similar sentences with a focus on changing tone, and body language. The complete dialogue can be found in Table S4. Body motion capture of the actor's movements to reconstruct the virtual mother was done with the Arena (https://www.naturalpoint.com/optitrack/products/arena/) Software, and facial animation recording using the Face shift studio Software (http://faceshift.com/studio/2015.2/). The final avatar was implemented in Motion Builder 2012 (http://www.autodesk.com/products/motionbuilder/overview) (Figure S1).

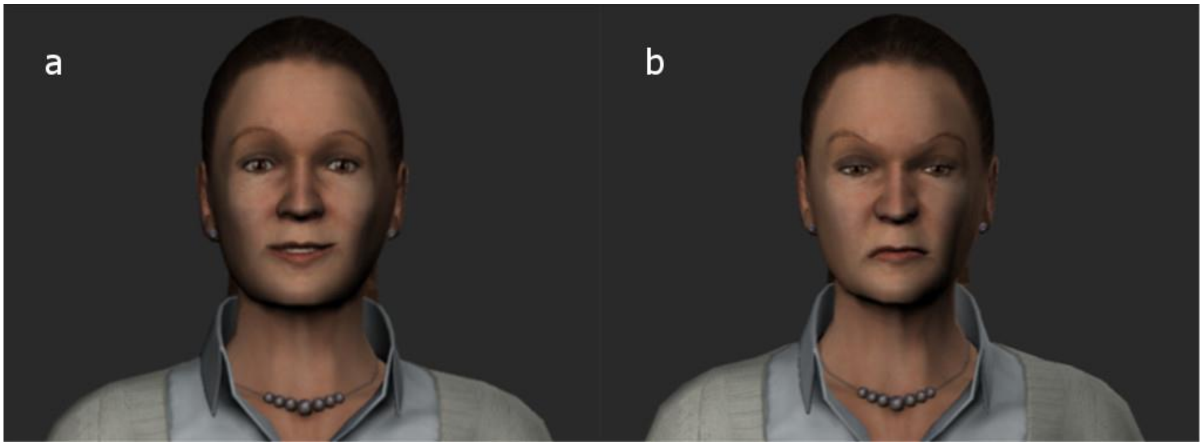

Figure S1. The mother avatar implemented with facial animation such as to reflect (a) the 'Positive' mother condition or (b) the 'Negative' mother condition.

Response Variables – Measurements. As indicated in the main text, in this experimental design four different types of response variables were used in order to measure participants' parenting style, empathy and level of embodiment in a child's body, as well as behavioural and physiological responses to the stimuli. Before the experiment, a demographics questionnaire was also used to record basic information about the participant such as age, status, number and age of children. Additional information, where relevant, is provided below. All questionnaires were available in English, Spanish and Catalan and used according to the preference of each participant.

The AAPI-2. Authors of the revised AAPI state that ach of the five parenting constructs of the AAPI-2 show diagnostic validity and can discriminate between the parenting behaviours of known abusive and non-abusive parents. Form A (pre-test) and Form B (post-test) both have 40 items, answered using a five point Likert Scale (Strongly Agree to Strongly Disagree). They also gather demographic data, plus experiences of childhood abuse and neglect. Time for completion is 10-15 minutes, fifth grade reading level and can be read aloud to participants with reading difficulties. Sub-scale responses are categorized as low, moderate or high risk for child maltreatment.

Participants' subjective experience. This 10-statement post-questionnaire assessing participants' subjective experience (Table S2) used a 7-point scale (− 3 Not at all to + 3 Very much). More specifically, these questions were related to the strength of body ownership (*MyBody, Mirror*) and agency (*Agency*), miscellaneous questions relating to the experience of being a child (*Younger*, *FeltChild*, *ChildLike*), while others served as control questions (*Features*, *TwoBodies*).

Mind in the Eyes. This was used to test affective empathy. The task involves describing the emotional state of a person based on only an image of their eyes. The task was presented pre and post intervention with the images appearing in random order. The presented images were photos of children's faces that were taken from the online NIMH-chEFS Picture Set (http://devepi.duhs.duke.edu/NIMH_Pictures.html), and only the eyes part was presented. Random whole face pictures were also mixed in the task in order to test the validity of the depicted emotions of the database when only the eyes were presented. The whole face images were not considered for the analysis.

***Physiological measurement*.** Participants were fitted with a g.Mobilab+ (multipurpose) (http://www.gtec.at/Products/Hardware-and-Accessories/g.MOBIlab-Specs-Features) physiological recording device that recorded heart activity ECG (256 Hz) and galvanic skin response GSR (256 Hz). Electrodes were placed on the palmar areas of the index and middle fingers of the non-dominant hand in order to record electrodermal activity. Electrodes were placed on the lowest left and right ribs and right collarbone in order to record ECG. The devices operate via Bluetooth. Both the online processing and the offline processing are controlled via Matlab (http://www.mathworks.es/index.html) and Simulink (http://www.mathworks.es/products/simulink/).

GSR serves an indication of psychological or physiological arousal through changes in skin conductance caused by skin moisture levels. An important derived measure of interest is the number of Skin Conductance Responses (SCR) which reflect transient sympathetic arousal, either spontaneous or in

response to events, specifically the orienting response, that is responses to changes in the environment and events or surprises. SCR response variable is defined to be the percentage of change between the maximum GSR amplitude on the baseline period and after each disruptive event. ECG is used to obtain a measure for the heart rate deceleration (HRD) in various stages of the experiment. HRD is a response variable significantly correlated with states of stress induced by sudden unpleasant stimuli, and it has been previously used in studies as a physiological correlate of the full body ownership illusion (Slater et al., 2010). ECG signals are processed to extract the heart rate (HR) by first applying the automatic search for QSR complexes in time series implemented in the g.tec biosignal analysis software g.BSanalyze (http://www.gtec.at/Products/Software/g.BSanalyze-Specs-Features), and then by visually inspecting the search results to correct for possible missing or false identifications.

Results

As noted in the main report, skin conductance response and heart rate deceleration showed effects of the negative mother, but the difference was not statistically significant (Figures S2 and S3 respectively).

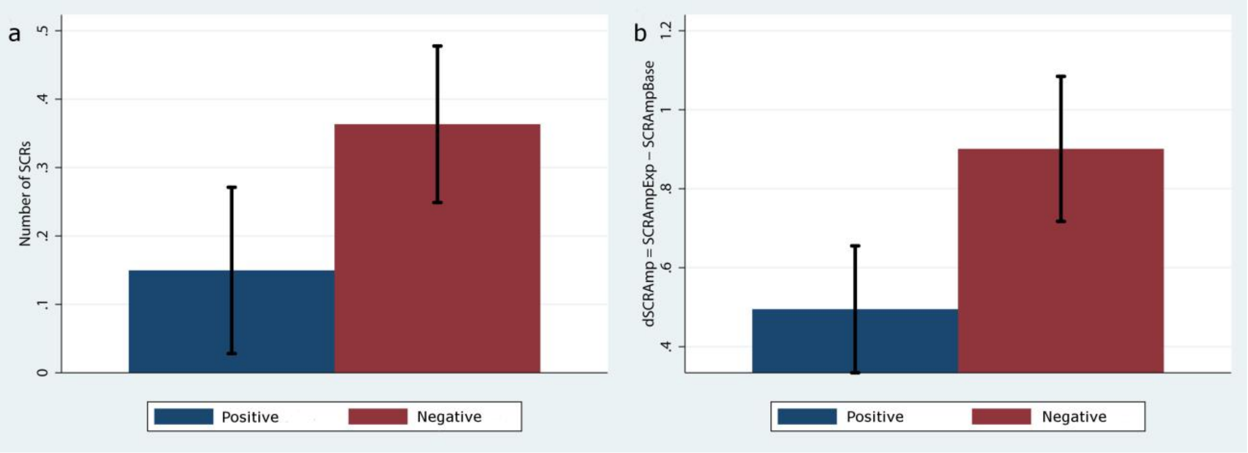

Figure S2. Main study: Bar charts for (a) number of SCRs by condition, and (b) mean dSCRAmp amplitude defined as SCRAmpExp – SCRAmpBase by condition. The heights are means and the bars SEMs. (Not statistically significant)

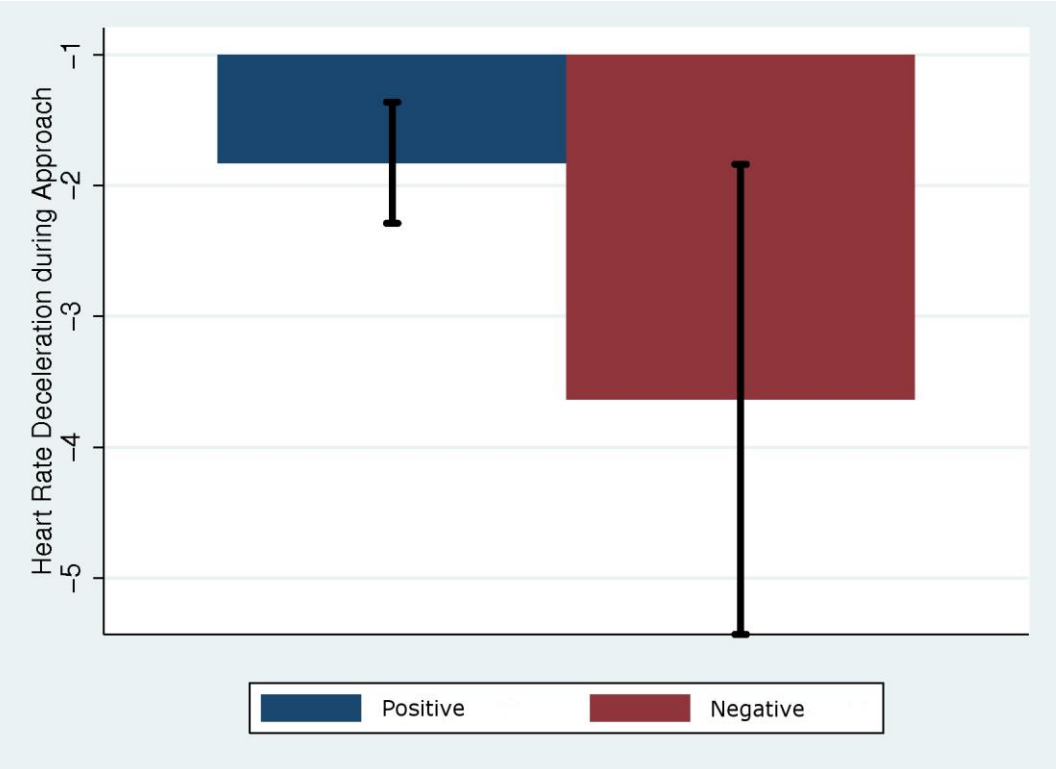

Figure S3. Main study: participants' HRD by condition for when the virtual mother approached the participant. The heights are means and the bars SEMs. (Not statistically significant.)

PILOT STUDY

Methods

Ethics. Ethical considerations were identical as in the main study.

Participants. Twelve Spanish mothers aged 33 to 45 years (mean age 38.5, SD=3.8) participated in the study. All were the biological mother of at least one child, some were also a stepmother to other children. In total, they had 23 children (mean 1.92 children, range 1-3) aged from 1 to 24 years. Of these, 14 were aged 1-5 years (60.9%), six were aged 6-9 years (26.1%), and three were aged 10 years or over (13%). In terms of education, 41.7% had completed college, 25% had some college, 25% were high school graduates and 8.3% (n=1) left after 11th grade. Over half were employed (41.7% part-time, 16.7% full-time) with the remaining 41.7% not employed/full-time mothers. In terms of abusive experiences, 75% stated that they had not experienced childhood abuse by a non-family member, the remaining

three participants declined to answer. In contrast, 25% reported abuse by a family member (n=3), one participant declined to answer (8.3%) and the rest reported no familial abuse (66.7%). None of the participants had prior experience in a virtual reality environment. All had normal (58.3%) or corrected (41.7%) vision.

Inclusion and exclusion criteria were identical as in the main study. Participants were paid €30 for participating (or a pro-rata rate if they chose to withdraw). All participants completed the study.

Design. The experimental design was identical as in the main study. The only difference was that participants experienced both conditions in the same setting (counter-balanced) with a 30 seconds break between the two interactions, without coming out of the VR system. One participant needed to stop part-way through the experiment due to dizziness; this occurred at the end of the first condition but after a break, she requested to continue, so the orientation phase was repeated followed by the other condition.

Procedures. Procedures were identical as in the main study. Participants experienced the same virtual settings, and interactions, with the difference that they experienced the two conditions during the same session. All embodiment, psychometric questionnaires and interview questions, as well as physiological data recordings were identical, except for the Mind in the Eyes test that was not included.

Environment. The virtual environment, virtual bodies as well as physical experimental laboratory, and equipment were the same as in the main study.

Results

Parenting Scale. In terms of parenting style (see Table S5), mean scores for this sample were comparable to published control group scores for Laxness (i.e., lower limit setting), published clinical group means for both Over-reactivity and Total score, but had higher mean scores than either group for Verbosity (i.e., amount of talking). This outcome could reflect cultural variations, but the Verbosity scale also has the lowest internal consistency. Initial analysis showed no significant relationships between

parenting styles and either pre or post intervention parenting scores, including empathy (as measured by the AAPI-2).

AAPI-2. The AAPI-2 gives five constructs. There was no significant difference pre-post on constructs A, C or D (expectations, corporal punishment and family roles; see Table S6). However, participant responses showed a significant improvement on Construct B (parental empathy for the child) with the mean score rising from 7.4 pre-intervention (medium risk) to 8.92 post-intervention (low risk; t=-3.593; p=.004; Figure S4). This sub-scale measures the parent's ability to understand and value children's needs, nurturance and positive growth, good communication and an ability to recognise the feelings of children. Notably, three high-scoring participants remained stable on this construct (two scored 8, one 10 on both occasions), but the other nine participants all recorded an improved empathy score. In contrast, Construct E also showed a significant difference, but this move actually reflected a change from valuing power and independence in children (7.42; medium risk) towards more of a tendency to restrict power and independence (5.58; medium risk; t=2.524, p=.028). This outcome, however, is likely to have been affected by one participant who gave an extreme response to this sub-scale that was out of context with her other responses (score of 10 pre – low risk, score of 1 post – high risk).

High empathy (pre) was positively correlated with appropriate expectations of a child (pre; r=.69, p=.01), appropriate family roles (pre; r=.57, p=.05), appropriate family roles (post; r=.71, p<.01). Post intervention high empathy was positively correlated with non-use of corporal punishment at both pre (r=.6, p<.05) and post (r=.6, p<.05), appropriate family roles (pre; r=.68, p=.01), values power and independence (pre; r=.64, p<.05).

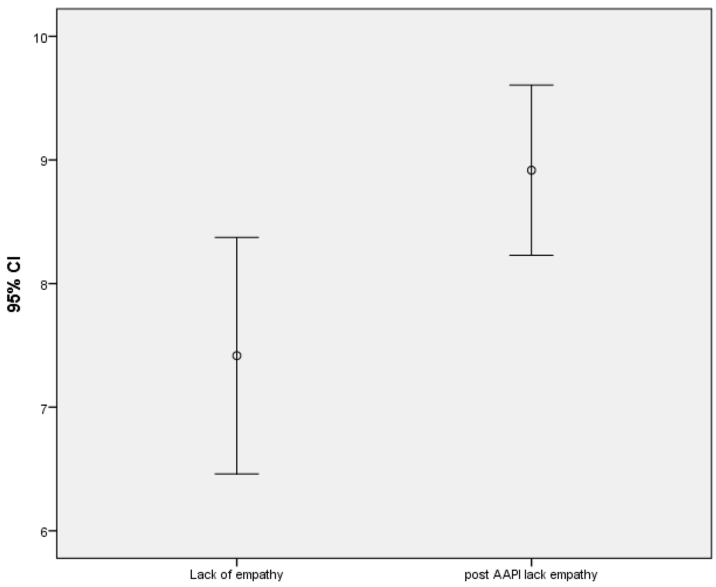

Figure S4. Pilot study empathy scores pre and post intervention rose from medium risk (lower score) to low risk (higher score).

Subjective experience responses. The extent to which individuals felt they were immersed in the system was explored. Frequency data is presented in Table S7.

Responses suggest that the majority of participants felt immersed in the environment, were responsible for making the movements, felt as if they were a child, and reacted in terms of thoughts, emotions and physically. There was no significant relationship between level of empathy (pre or post; AAPI-2) and any of the subjective experience questions.

Physiological data. For some participants there were peaks of higher heart rate or skin response with angry mother. In condition one (Positive-Negative), the pattern of heart rate remained quite similar (Figure S5a). In condition two (Negative, Positive), there was more tendency for rises in heart rate with the angry mother that also remained higher with the nice mother (Figure S5b). The pattern was similar regarding SCR (Figure S6).

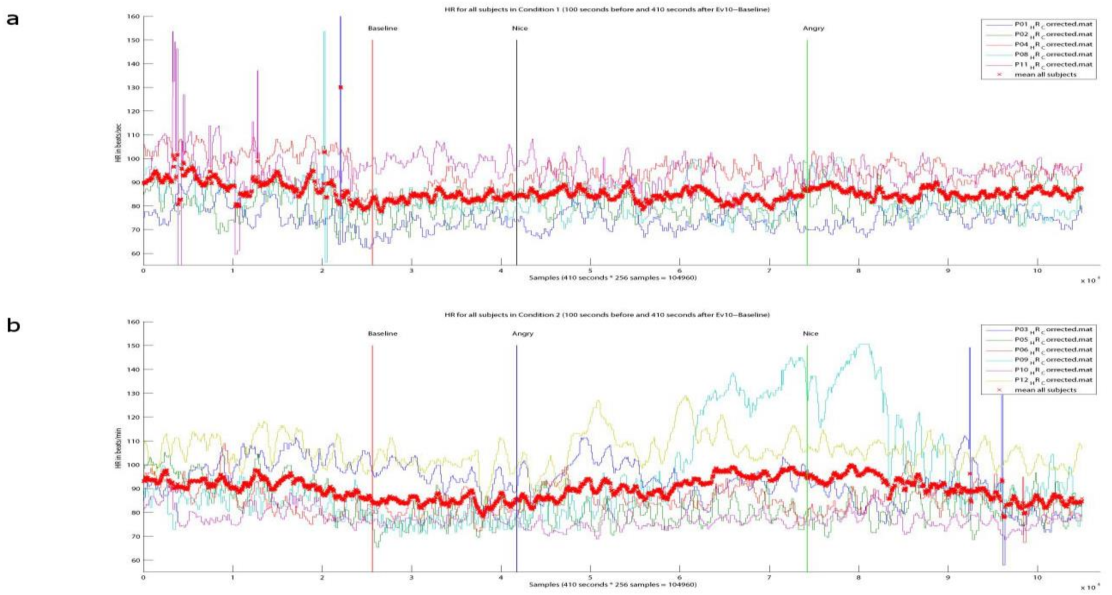

Figure S5. Pilot study heart rate for participants in (a) condition 1 (Positive-Negative), and (b) condition 2 (Negative-Positive)

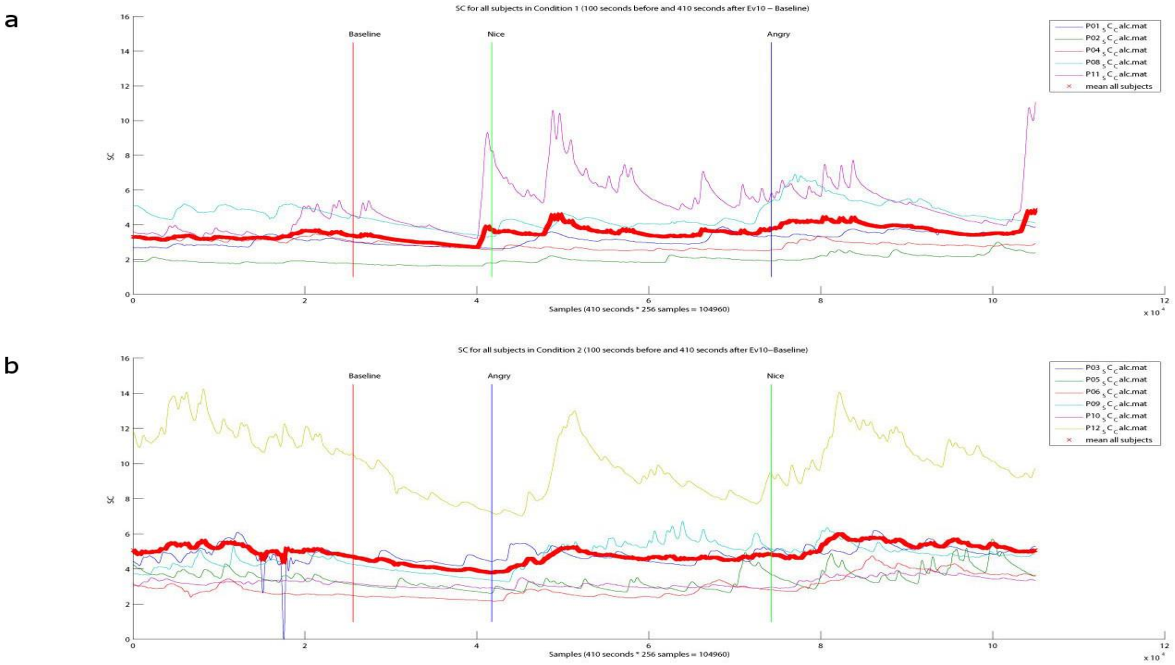

Figure S6: Pilot study galvanic skin response in (a) condition 1 (Positive-Negative), and (b) condition 2 (Negative-Positive).

Tables

Table S1. The experimental design. Distribution of participants by condition, showing that the experimental groups were comparable across a number of variables.

	Positive-Negative	Negative-Positive
**Female**	n = 10	n = 10
**Mean ± S.E Age**	40±1.06	38.7±1.49
**Median Code Previous VR Experience (IQR)**	1(0)	1(0)
**Median Code Games (IQR)**	1(1)	3.5(4)
**Mean ± S.E Children**	1.5±0.22	1.5±0.22
**Mean ± S.E Child Age**	7.6±1.21	5.2±1.18
**Median Code Education (IQR)**	10(0)	9.5(2)
**Median Code Employment (IQR)**	2(1.5)	2.5(2)
**Median Code Laxness (IQR)**	2.63(0.54)	2.68(0.9)
**Median Code Over reactivity (IQR)**	2.95(0.7)	2.8(0.2)
**Median Code Verbosity (IQR)**	4.35(1)	4.57(1.07)

*Groups Positive-Negative and Negative-Positive are formed based on the condition which each participant was assigned to during the first trial: interaction with caring mother (Positive) or angry mother (Negative). For each case the total number of participants, mean of ages, median and IQR values for participants' experience in VR and hours of video games are given. Codes refer to a 1 – 7 Likert scale. For previous VR experience 1 means the least and 7 the most experience, and for hours spent playing video games 1 means least and 7 most.

Table S2. Post Experience Questionnaire Items. All questions were scored on a -3 to +3 scale, where -3 meant least and +3 meant most agreement with the statement.

Variable Name	Question	Scoring Scale
MyBody	'How much did you feel that the virtual body you were looking at was your own (real) body?'	Not at all/ Very much (-3…3)
Mirror	'How much did you feel that the virtual body you were looking at in the mirror was your own (real) body?'	Not at all/ Very much (-3…3)
Features	'How much did you feel that the virtual body resembled your own (real) body in terms of shape, skin tone or other visual features?'	Not at all/ Very much (-3…3)
TwoBodies	'How much did you feel as if you had two bodies?'	Not at all/ Very much (-3…3)
Agency	'How much did you feel that the movements of the virtual body were caused by your own movements?'	Not at all/ Very much (-3…3)
MotherPresence	'How much did you feel that the virtual woman you saw was conscious of your presence?'	Not at all/ Very much (-3…3)
MotherAssaulted	'Have your thought at any moment that the virtual woman you saw could be physically assaulted during the experience?	Not at all/ Very much (-3…3)
RealSituation	'How much did you feel the experience as if it were real?'	Not at all/ Very much (-3…3)

Thoughts	'How much did you find yourself responding to the virtual woman regarding your thoughts?'	Not at all/ Very much (-3…3)
Emotions	'How much did you find yourself responding to the virtual woman regarding your emotions?'	Not at all/ Very much (-3…3)
Physical	'How much did you find yourself responding to the virtual woman regarding your physical reaction?'	Not at all/ Very much (-3…3)
Childlike	'How much did the virtual woman act as if you were a child?'	Not at all/ Very much (-3…3)
Violence	'How violent did you find the scene?'	Not at all/ Very much (-3…3)
Assaulted	'Have you felt at any moment that you could be physically assaulted during the experience?'	Not at all/ Very much (-3…3)
Younger	'How much younger than your real age did you feel?'	Not at all/ Very much (-3…3)
FeltChild	'How much did you feel like a child?'	Not at all/ Very much (-3…3)

Table S3. Pre, during and post-experiment measurements.

**Pre-measures**	1. Demographic details, including number and age of children**)**<br>2. Parenting scale ((Arnold, O'Leary, Wolff, & Acker, 1993); Spanish or Catalan version) – to assess discipline practices of parents:<br>• Over-reactivity<br>• Verboseness<br>• Laxness<br>• Total score<br>3. Mind in the Eyes test (Baron-Cohen et al., 2001)<br>4. Adult Adolescent Parenting Inventory – version 2 (AAPI-2; Bavolek & Keene, 2001) – Form A; to assess parenting and child rearing attitudes (Spanish translation and norms available). Measure of risk in:<br>1. **Construct A** - Expectations of Children<br>2. **Construct B** - Parental Empathy towards Children's Needs<br>3. **Construct C** - Use of Corporal Punishment<br>4. **Construct D** - Parent-Child Family Roles<br>5. **Construct E** - Children's Power and Independence
**During**	1. Physiological measurements (i.e., heart rate and galvanic skin response) – data was recorded for a baseline relaxation period of 120 seconds (Baseline), and during the interaction with the virtual avatar for another 120 seconds (Interaction).

**Postmeasures**	1. Qualitative semi-structured interview:<br><br>• How was that experience?<br>• How did you feel when the mother was so angry/negative?<br>• How did you feel when the mother was warm?<br>• Do you think that this experience will make you **think** more about how you respond to your own children? Why?<br>• Do you think that this experience will make you **behave** differently to your own children? Why?<br><br>2. Measure of subjective experience in the virtual environment.<br><br>• A 16-statement questionnaire<br>• 7-point scale was used ranging from '– 3' ('Strongly Disagree') to '+ 3' to ('Strongly Agree').<br>• Questions on strength of body ownership (Q1, Q2) and agency (Q5), miscellaneous questions relating to the experience of being a **child** (Q6-Q10, Q12, Q15-Q16), while others served as control questions (Q3, Q4).<br><br>3. Quantitative measure: AAPI-2 (Bavolek & Keene, 2001) – Form B; post intervention.<br><br>4. Mind in the Eyes test

Table S4. The interaction script.

Main Dialogue plus back up phrases

**Condition 'Positive'**	**Condition 'Negative'**
[*Mother*]: Hi darling how are you?	[*Mother*]: There you are, what are you doing?
[*Mother*]: You are not bored, are you?	[*Mother*]: Did you call me? I am sure I heard you saying something
[*Mother*]: Do you want to play together for a while?	[*Mother*]: don't bother me; can't you see I am busy?
[*Mother*]: Come on, what toy do you like the most?	[*Mother*]: Don't you like any of these toys?
[*Mother*]: That is a very nice toy, I like it too. Tell me what is it that you like about this toy?	[*Mother*]: You are making too much noise! Stop making noise.
[*Mother*]: You are right. Um, I'll go and start making dinner and then perhaps we can go for a little walk together - we are going to have fun! [Back up phrase: don't you want to come out for a walk with me?]	[*Mother*]: Look, I'm going to make dinner and then we are out of here. I've had enough being inside here.
[*Mother*]: Well, then I'll see you in a bit?	

[*Mother*]: In the meantime, would you mind tiding up the room here a bit to help me out, please? I am very tired and that would be such a big help for me.	*Mother*]: Once I go, tidy up this room. You have left everything untidy. The room looks a disaster. For God's sake, why can't you do what I am asking you for once? I am tired of doing everything around here without help and on top of that it's you who disorganizes everything, who doesn't do anything. What are you looking at? Tidy up! Now
[[*Mother*] What a sweet girl you are! I love you so much! You always help me out, I'm very proud of you and I love spending time together. So does your daddy, maybe when he comes back he can be with us and we can play a game together.	[*Mother*]: You are such an untidy child. Why do I have such a naughty daughter? Why don't you do what I ask for once? Is it too much to ask to leave me in peace now and then? Leave me in peace! Really, you are such a stupid girl, just like your father, stupid. Wait until he gets home and he hears how horrible you've been today, you will see!

Table S5. Mean scores for parenting styles for the Spanish participants (pilot study; N=12) and normative data

	**Spanish group (N=12)**		**Norms***	
			**Clinical (N=26)**	**Control (N=51)**
	**Mean (SD)**	**range**	**Mean (SD)**	**Mean (SD)**
**Mother's age**	38.5 (SD=3.8)	33-45	29.6 (6.7)	31.7 (3.9)
**Laxness**	2.36 (0.7)	1.18-3.36	2.8 (1.0)	2.4 (0.8)
**Over-reactivity**	3.00 (0.67)	2.00-4.40	3.0 (1.0)	2.4 (0.7)
**Verbosity**	4.08 (0.66)	3.14-5.57	3.4 (1.0)	3.1 (1.0)
**Total score**	3.13 (0.44)	2.53-4.07	3.1 (1.7)	2.6 (0.6)

**O'Leary, Arnold, Wolff, & Acker (1993)*

Table S6. Pilot study AAPI-2 scores pre and post scenarios (N=12)*

	**Pre M (SD)**	**Post M (SD)**	**T**	**Significance**
**Construct A** Expectations of Children	7.75 (1.4)	7.5 (1.6)	.897	.389
**Construct B** Parental Empathy towards Children's Needs	7.42 (1.5)	8.92 (1.1)	-3.593	**.004**
**Construct C** Use of Corporal Punishment	8.00 (1.5)	8.1 (1.4)	-.321	.754
**Construct D** Parent-Child Family Roles	9.5 (0.8)	9.6 (0.5)	-.432	.674
**Construct E** Children's Power and Independence	7.42 (1.6)	5.58 (2.5)	2.524	**.028**

*range of scores 1-10; a high score is positive, low risk; low scores of 3 or under are negative and indicate high risk

Table S7. Pilot study subjective experiences reported by participants (N=12).

	Not agree		Neutral		Agreed	
	n	%	n	%	n	%
Virtual body was own	1	8.3	1	8.3	10	83.4
Looking at self in mirror	2	16.6	-		10	83.4
VR resembled own body, face, skin etc.	5	41.7	2	16.7	5	41.7
Felt as if had two bodies	8	66.7	-		4	33.3
VR movement caused by own body movement	-		1	8.3	11	91.7
VR mother aware of you	-		-		12	100.0
Responded to mother - thoughts	2	16.6	1	8.3	9	75.1
Responded to mother - emotionally	2	16.6	-		10	83.4
Responded to mother - physically	4	33.3	-		8	66.7
VR mother acted towards you as a child	-		2	16.7	10	83.4
Felt like a child	1	8.3	1	8.3	10	83.4
